# Interventions to reduce suicides at suicide hotspots: a systematic review

**DOI:** 10.1186/1471-2458-13-214

**Published:** 2013-03-09

**Authors:** Georgina R Cox, Christabel Owens, Jo Robinson, Angela Nicholas, Anne Lockley, Michelle Williamson, Yee Tak Derek Cheung, Jane Pirkis

**Affiliations:** 1 Orygen Youth Health Research Centre, Centre for Youth Mental Health, University of Melbourne, Melbourne, Australia; 2 University of Exeter Medical School, Exeter, UK; 3 Centre for Health Policy, Programs and Economics, Melbourne School of Population and Global Health, University of Melbourne, Melbourne, Australia

**Keywords:** Suicide hotspots, Suicide prevention, Intervention

## Abstract

**Background:**

‘Suicide hotspots’ include tall structures (for example, bridges and cliffs), railway tracks, and isolated locations (for example, rural car parks) which offer direct means for suicide or seclusion that prevents intervention.

**Methods:**

We searched Medline for studies that could inform the following question: ‘*What interventions are available to reduce suicides at hotspots, and are they effective?’*

**Results:**

There are four main approaches: (a) restricting access to means (through installation of physical barriers); (b) encouraging help-seeking (by placement of signs and telephones); (c) increasing the likelihood of intervention by a third party (through surveillance and staff training); and (d) encouraging responsible media reporting of suicide (through guidelines for journalists). There is relatively strong evidence that reducing access to means can avert suicides at hotspots without substitution effects. The evidence is weaker for the other approaches, although they show promise.

**Conclusions:**

More well-designed intervention studies are needed to strengthen this evidence base.

## Background

A ‘suicide hotspot’ is a specific, accessible and usually public site which is frequently used as a location for suicide and gains a reputation as such [1]. The most common types of suicide hotspot are bridges, tall buildings and cliffs [2-5], railway tracks [6,7], and rural or secluded locations [8]. The suicide methods typically used at these sites, such as jumping from a height, jumping or lying in front of a train and inhalation of car exhaust have a high probability of being lethal [9]. Suicides at hotspots can have a distressing impact on those who witness the event, find the deceased or are involved in some other way [10,11]. They often receive high profile media coverage [12], which may increase the risk of ‘suicide contagion’. There is no agreement on the number of suicides that is required to identify a site as a ‘suicide hotspot’. However, more than one suicide at a particular location suggests that the site has appeal for suicidal individuals and provides means or opportunity for suicide, and may therefore warrant intervention [13].

Various interventions have been implemented to reduce the risk of suicide at suicide hotspots. The current review examines the evidence for the effectiveness of these interventions. Specifically, it addresses the following research question: ‘*What interventions are available to reduce suicides at hotspots, and are they effective?’*

## Methods

**Figure 1:**
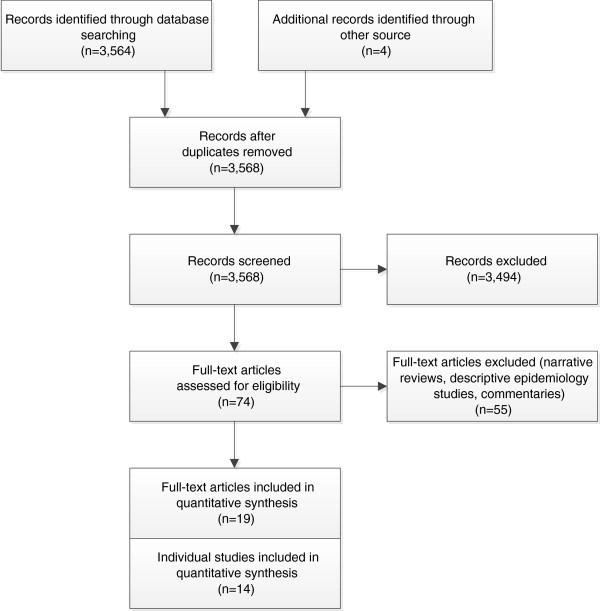
PRISMA flow diagram.

Our review was conducted in accordance with the Preferred Reporting Items for Systematic Reviews and Meta-Analyses (PRISMA) Statement [14] (see Figure 1 and Table 1).

**Table 1:** PRISMA checklist

**SECTION/TOPIC**	**#**	**CHECKLIST ITEM**	**REPORTED ON PAGE #**
**TITLE**			
Title	1	Identify the report as a systematic review, meta-analysis, or both.	0
**ABSTRACT**			
Structured summary	2	Provide a structured summary including, as applicable: background; objectives; data sources; study eligibility criteria, participants, and interventions; study appraisal and synthesis methods; results; limitations; conclusions and implications of key findings; systematic review registration number.	1
**INTRODUCTION**			
Rationale	3	Describe the rationale for the review in the context of what is already known.	1
Objectives	4	Provide an explicit statement of questions being addressed with reference to participants, interventions, comparisons, outcomes, and study design (PICOS).	1
**METHODS**			
Protocol and registration	5	Indicate if a review protocol exists, if and where it can be accessed (e.g., Web address), and, if available, provide registration information including registration number.	N/A
Eligibility criteria	6	Specify study characteristics (e.g., PICOS, length of follow-up) and report characteristics (e.g., years considered, language, publication status) used as criteria for eligibility, giving rationale.	2,4
Information sources	7	Describe all information sources (e.g., databases with dates of coverage, contact with study authors to identify additional studies) in the search and date last searched.	1
Search	8	Present full electronic search strategy for at least one database, including any limits used, such that it could be repeated.	1-2
Study selection	9	State the process for selecting studies (i.e., screening, eligibility, included in systematic review, and, if applicable, included in the meta-analysis).	2
Data collection process	10	Describe method of data extraction from reports (e.g., piloted forms, independently, in duplicate) and any processes for obtaining and confirming data from investigators.	2,4
Data items	11	List and define all variables for which data were sought (e.g., PICOS, funding sources) and any assumptions and simplifications made.	2,4
Risk of bias in individual studies	12	Describe methods used for assessing risk of bias of individual studies (including specification of whether this was done at the study or outcome level), and how this information is to be used in any data synthesis.	11
Summary measures	13	State the principal summary measures (e.g., risk ratio, difference in means).	4
Synthesis of results	14	Describe the methods of handling data and combining results of studies, if done, including measures of consistency (e.g., I^2^) for each meta-analysis.	4
Risk of bias across studies	15	Specify any assessment of risk of bias that may affect the cumulative evidence (e.g., publication bias, selective reporting within studies).	11
Additional analyses	16	Describe methods of additional analyses (e.g., sensitivity or subgroup analyses, meta-regression), if done, indicating which were pre-specified.	N/A
**RESULTS**			
Study selection	17	Give numbers of studies screened, assessed for eligibility, and included in the review, with reasons for exclusions at each stage, ideally with a flow diagram.	2
Study characteristics	18	For each study, present characteristics for which data were extracted (e.g., study size, PICOS, follow-up period) and provide the citations.	5-8
Risk of bias within studies	19	Present data on risk of bias of each study and, if available, any outcome level assessment (see item 12).	N/A
Results of individual studies	20	For all outcomes considered (benefits or harms), present, for each study: (a) simple summary data for each intervention group (b) effect estimates and confidence intervals, ideally with a forest plot.	5-8
Synthesis of results	21	Present results of each meta-analysis done, including confidence intervals and measures of consistency.	N/A
Risk of bias across studies	22	Present results of any assessment of risk of bias across studies (see Item 15).	N/A
Additional analysis	23	Give results of additional analyses, if done (e.g., sensitivity or subgroup analyses, meta-regression [see Item 16]).	N/A
**DISCUSSION**			
Summary of evidence	24	Summarize the main findings including the strength of evidence for each main outcome; consider their relevance to key groups (e.g., healthcare providers, users, and policy makers).	4,9
Limitations	25	Discuss limitations at study and outcome level (e.g., risk of bias), and at review-level (e.g., incomplete retrieval of identified research, reporting bias).	11
Conclusions	26	Provide a general interpretation of the results in the context of other evidence, and implications for future research.	11
**FUNDING**			
Funding	27	Describe sources of funding for the systematic review and other support (e.g., supply of data); role of funders for the systematic review.	11

### Search strategy

We searched Medline from its inception to April 2012 using the following search string, with words mapped onto MeSH headings: (suicid* OR hotspot) AND (cliff OR building OR high-rise OR multi-storey OR viaduct OR rail OR metro OR subway OR river OR lake OR sea OR public* OR secluded OR remote OR woods OR forest OR rural OR magnet OR location OR secluded OR bridge OR skyscraper OR car park OR underground OR road OR motorway OR highway OR reservoir OR coast OR jump* OR leap* OR fall OR height OR lie OR lying OR moving object OR carbon monoxide OR car exhaust OR hang* OR firearm OR gun* OR burn* OR drown* OR fenc* OR barrier* OR parapet OR net* OR pit* OR sign* OR poster* OR helpline* OR surveillance* OR CCTV OR patrol* OR media OR reporting OR television OR radio. Reference lists of key review papers and included studies were also searched. We sought only English-language publications.

### Study inclusion criteria

We gave careful consideration to the kinds of studies that we would include in the review. Hotspot interventions are generally offered as universal preventive strategies, rather than as preventive strategies targeted at individuals. This means that evaluations usually draw on aggregate or ecological data on completed or attempted suicide, rather than individual-level data. It is practically and ethically difficult to mount cluster randomised controlled trials (cRCTs, regarded as Level I evidence) in this area. Suicide is an emotive issue and suicide hotspots generate considerable community concern, which means that randomly selecting some sites to receive the intervention is generally not feasible. Ecological studies with quasi-experimental designs (non-randomised studies with before-and-after designs and comparison sites, regarded as Level II evidence), are the next best solution, but comparable sites are not always available (for example, where one bridge is a recognised hotspot and nearby bridges do not present the same problem). This means that ecological studies with before-and-after designs and no comparison sites (regarded as Level III evidence) are often the most acceptable and appropriate option in the circumstances.

We included studies in the review if they described an intervention relating to a known suicide hotspot, evaluated it using at least a before-and-after design with no comparison (but preferably a stronger design), and used suicides as the outcome of interest (with or without other outcomes, such as suicide attempts). Studies which only measured suicides after an intervention was put in place and/or only considered outcomes other than suicides (such as calls to a crisis telephone service) were excluded.

### Data extraction

The following data were extracted for each study:

•Author(s) and date of publication;

•Setting;

•General approach(es);

•Specific intervention(s);

•Study design and observation period; and

•Findings.

With respect to the findings, data were examined on changes in the number or rate of suicides at the hotspot site and at comparison sites where these were available. Consideration was also given to whether there was any evidence of substitution, either in the form of site substitution (reduction in suicides at the hotspot being accompanied by increases at other nearby sites) or method substitution (reduction in use of one method being accompanied by increases in others).

## Results

Nineteen papers describing 14 studies at 13 locations worldwide met our inclusion criteria [15-33], and are summarised in Table 2. There were several instances where the same group of authors used the same core data in more than one paper, augmenting it with data from other sources or with follow-up data [15-20,26,32]. In these cases, we took the conservative approach of regarding the different papers as relating to the same study to avoid double-counting of any observed impacts, thereby circumventing the possibility of multiple publication bias. There was one instance in which the same data were examined independently by separate investigators to determine the impact of the same intervention at the same site [24,27]. These were regarded as separate studies, but their findings are discussed together, again to avoid artificially inflating the collective magnitude of any impact.

**Table 2:** Study characteristics and results

**Author(s) and date**	**Study number**	**Setting**	**General approach(es)**	**Specific intervention(s)**	**Study design and observation period**	**Findings**
Beautrais (2001) [15]; Beautrais et al. (2009) [16]	1	Grafton Bridge, Auckland, New Zealand.	• Restricting access to lethal means	Metal screens fixed above concrete parapets for purposes of suicide prevention, removed in 1996. Reinstallation of a barrier in 2003 with an improved curved glass design.	A-B-A (reversal) study assessing number and rates of suicides in three periods:	• Five suicides from the bridge during period in which original barriers were in place (1.0 per year). This rose to 19 in period when original barriers were removed (3.2 per year). No suicides occurred after the installation of the new barrier.
					• 1991–1995 (5-year period in which original barriers were in place);	
					• 1997–2002 (5 year period in which no barriers were in place); and	
						• No change in overall number of suicides by jumping in Auckland.
					• 2003–2006 (5 year period in which new barriers were in place).	
Bennewith, Nowers and Gunnell (2007) [17]; Bennewith, Nowers and Gunnell (2011) [18]	2	Clifton Suspension Bridge, Bristol, United Kingdom.	• Restricting access to lethal means	Two metre high wire fencing installed on main span in 1998.	Before-and-after analysis comparing number of suicides in two periods:	• Suicides dropped from 8.2 per year in period prior to installation of fencing to 4.0 per year in period following it.
			• Increasing the likelihood of intervention by a third party	Role of bridge staff expanded to include ensuring individuals’ safety and monitoring incidents. CCTV cameras installed.	• 1994–1998 (5-year pre-intervention period); and	
						• 90% of suicides from the bridge were by males but there was no evidence of an increase in male suicide by jumping from other sites in Bristol following the installation.
					• 1999–2003 (5-year post-intervention period).	
					Before-and-after analysis comparing number of suicides in two periods:	
					• 1996–1998 (3-year pre-intervention period); and	• Number of incidents remained stable (39 per year in pre-installation period; 43 per year in post-installation period).
					• 1999–2005 (7-year post-intervention period).	
						• Bridge staff more likely to be involved in incidents after the installation of barriers.
					Interviews with 10 of 13 bridge staff.	• Majority of interviewed bridge staff felt that the barriers had been successful in preventing suicide.
Etzersdorfer and Sonneck (1992) [19]; Sonneck, Etzersdorfer and Nagel-Kuess (1994) [32]; Etzersdorfer and Sonneck (1998) [20]; Niederkrotenthaler and Sonneck (2007) [26]	3	Vienna underground railway system, Vienna, Austria	• Providing guidance on responsible media reporting of suicide	Guidelines on media reporting of suicides – with particular reference to railway suicides – developed and disseminated.	Before-and-after analysis comparing number of completed and attempted railway suicides in two periods:	• Suicidal acts on the underground railway system rose dramatically in the latter part of the pre-intervention period (when sensationalist reports of suicide were common), peaking at nine completed suicides and 10 attempted suicides in the first half of 1987. Following the introduction of the guidelines, both completed and attempted suicides dropped dramatically (to two and one incidents, respectively). This level was then sustained for the remainder of the observation period.
					• 1 Jan 1980 – 30 June 1987 (7.5 year pre-intervention period); and	
					• 1 July 1987 – 31 Dec 1996 (9.5 year post-intervention period).	
					Subsequent interrupted time series analysis that examined trends in overall suicides from 1946/47 to 2004/05 and trends in railway suicides from 1982/83 to 2004/05.	
						• Some evidence of nationwide impact, with a reduction of 81 overall suicides.
Isaac and Bennett (2005) [22]	4	Beachy Head, Sussex, United Kingdom	• Restricting access to lethal means	Road access blocked from Jan-Jun 2001 due to foot and mouth crisis	Before-and-after analysis comparing number of suicides in two periods:	• Suicides had risen to a high in the pre-intervention period (85% higher than in 1965–1979) but reduced to zero once road access was blocked.
					• 1987–2000 inclusive (14-year pre-intervention period); and	
					• Jan-Jun 2001 (6-month post-intervention period).	
Law et al. (2009) [23]	5	Hong Kong underground railway system, Hong Kong. This system is operated by the Mass Transit Railway (MTR) Corporation and the Kowloon-Canton Railway (KCR) Corporation.	• Restricting access to lethal means	Platform Screen Doors (PSDs) installed on 71 platforms in 30 MTR underground stations on three prominent transit lines. Work began in 2002 and ended in 2005, but most of the busiest station platforms were sealed in the first year.	Before-and-after analysis comparing number of suicides in two periods.	• Significant decrease in the number of suicides on the Hong Kong underground railway system from 51 (10.2 per year) in the pre-installation period to 22 (4.4 per year) in the post-installation period.
					• 1997–2001 (5-year pre-intervention period); and	
					• 2003–2007 (5-year post-intervention period).	
						• No evidence for displacement to other rail platforms; the number of suicides at MTR stations dropped from 38 to seven, whereas the number at KCR stations remained fairly stable (13 in the pre-installation period and 15 in the post-installation period).
					Incorporated quasi-experimental design element which considered numbers of suicides over time at stations with and without PSDs.	
King and Frost (2005) [21]	6	New Forest, Hampshire, United Kingdom	• Encouraging help-seeking	Signs displaying Samaritans’ national telephone number placed in 26 car parks in 1998.	Before-and-after analysis comparing number of suicides in two periods:	• Car park suicides dropped from 10.0 per year in period prior to installation of signs to 3.3 per year in the period following it.
					• 1 Oct 1988 – 30 Sept 1998 (10-year pre-intervention period); and	
						• Average annual total number of suicides in the district also decreased.
					• 1 Oct 1998 – 30 Sept 2001 (3-year post-intervention period).	
						• No changes were found in comparable forest districts.
					Incorporated quasi-experimental design element which considered numbers of suicides in same periods in comparable forest districts.	
Lester (1993)[24]; O’Carroll and Silverman (1994) [27]	7, 8	Ellington Bridge, Washington DC, United States	• Restricting access to lethal means	Eight foot fence installed in 1986.	Before-and-after analysis comparing number of suicides in two periods:	• Suicides dropped from 3.7 per year in period prior to installation of fencing to 0.2 per year in period following it.
					• 1979–1985 (7-year pre-intervention period); and	
						• Suicides from nearby Taft Bridge remained relatively stable (1.7 in pre-installation period; 2.0 in post-installation period).
					• 1986–1990 (5-year post-intervention period).	
						• Overall mean number of suicides in Washington DC was 76.4 in the pre-installation period and 71.6 in the post-installation period.
Lester (2005) [25]	9	Sunshine Skyway Bridge, St Petersburg, Florida, United States	• Encouraging help-seeking	Crisis emergency telephones installed in 1999 and a police presence on the bridge established at the same time.	Before-and-after analysis comparing number of suicides in two periods:	• Suicides dropped from 25 in pre-intervention period (8.3 per year) to 19 in post-intervention period (6.3 per year).
					• 1996–1998 (3-year pre-intervention period); and	
					• 2000–2002 (3-year post-intervention period).	
			• Increasing the likelihood of intervention by a third party			
Pelletier (2007) [28]	10	Memorial Bridge, Augusta, Maine, United States.	• Restricting access to lethal means	11 foot high fence installed on either side bridge in 1983.	Before-and-after analysis comparing number of suicides in two periods:	• 14 suicides in period prior to installation of fence; none in period following installation.
					• 1 Apr 1960 – 31 May 1983 (22-year pre-intervention period); and	
						• Number of suicides by jumping or drowning at sites in Augusta other than the Memorial Bridge remained unchanged (nine in each period).
					• 1 Jun 1983 – 31 Jul 2005 (22-year post-intervention period).	
						• Overall suicide rate in Augusta dropped by 9.0% (from 26.0/100,000 in pre-installation period to 23.8 per 100/000 in post-installation period).
Reisch and Michel (2005) [29]	11	Muenster Terrace, Bern, Switzerland.	• Restricting access to lethal means	Four metre wide wire mesh net, 7 metres below top of terrace installed in 1998 following high level of media attention.	Interrupted time series analysis assessing expected and observed numbers of suicides in two periods:	• No suicides from the terrace in the period following installation of safety net.
						• Overall decrease in suicides by jumping from all sites in Bern (95 expected; 44 observed).
					• 1995–1998 (4-year pre-intervention period); and	
					• 1999–2002 (4-year post-intervention period)	
Sinyor and Levitt (2010) [30]	12	Bloor Street Viaduct, Toronto, Canada	• Restricting access to lethal means	Five metre high barrier constructed between April 2002 and June 2003 comprising closely-spaced steel rods supported by an angled steel frame.	Before-and-after analysis comparing number of suicides in pre- and post-intervention periods:	• Annual numbers of suicide from the viaduct dropped from 9.3 to 0.0 after the barrier was installed.
					• 1993–2001 (9-year pre-intervention period); and	• No reduction in overall rates of suicide by jumping due to increase in suicides by this method at other Toronto sites.
					• 2003–2007 (5-year post-intervention period).	
Skegg and Herbison (2009) [31]	13	Lawyers Head Cliff, Dunedin, New Zealand	• Restricting access to lethal means	Road access blocked in 2006 due to maintenance.	Before-and-after analysis comparing number of suicides in two periods:	• 14 deaths in the 10-year period before closure (11 suicides, two open verdicts and one accidental death); none during 2-year closure period.
					• 1 Aug 1996 – 31 Jul 2006 (10-year pre-intervention period); and	
					• 1 Aug 2006 – 31 Jul 2008 (2-year post-intervention period).	• 77 police call outs for threatened or attempted suicide in 4-year period before closure (19.3 per year); 19 call-outs during closure period (9.8 per year).
					Before-and-after analysis comparing number of police call-outs in two periods:	
					• 1 Aug 2002 – 31 Jul 2006 (4-year pre-intervention period); and	
					• 1 Aug 2006 – 31 Jul 2008 (2-year post-intervention period).	
Wong et al. (2009) [33]	14	Cheung Chau, Hong Kong. This is an island which attracted visitors who rented holiday flats in which they took their own lives by charcoal burning.	• Encouraging help-seeking	Integrative suicide prevention program established in 2002 which included telephone hotlines, gatekeeper training and suicide patrols.	Before-and-after analysis comparing number of completed and attempted suicides in two periods:	• Visitor completed suicides dropped from 37 (8.7 per year) in pre-intervention period to 6 (2.0 per year) in post-intervention period.
			• Increasing the likelihood of intervention by a third party			
					• 1 Jan 1998 – 31 Mar 2002 (4.25 year pre-intervention period); and	
						• Visitor attempted suicides remained relatively stable (27 (6.4 per year) in pre-intervention period; 24 (6.9 per year) in post-intervention period).
					• 1 Oct 2002 – 31 Mar 2006 (3.50 year post-intervention period).	
					Incorporated quasi-experimental design element which considered numbers of completed and attempted suicides in same periods on two islands with similar demographic profiles.	• No comparable change in visitor suicides on comparison islands over study period.

The interventions studied fall into four broad categories representing different approaches to suicide prevention. The most commonly-investigated of these is restricting access to means (for example, by installing barriers at a jumping site). The second approach involves encouraging help-seeking (for example, via signs and telephone crisis lines). The third intervention involves increasing the likelihood of intervention by a third party (for example, offering training for staff working at or near suicide hotspots and/or surveillance methods). The final approach is the provision of guidance on responsible reporting of suicide to media professionals, in order to minimise the risk of ‘suicide contagion’ at hotspots. Most of the studies consider a single intervention, but some consider several together.

### Restricting access to means

Nine studies have examined the effectiveness of restricting access to lethal means by installing physical barriers at sites that are used for jumping from a height or jumping in front of a train. All of these studies suggest that suicides reduce once means restriction measures are put in place [15-18,22-24,27-31], or rise when they are removed [15,16].

Pelletier [28], Reisch and Michel [29] and Sinyor and Levitt [30] observed no further suicides after barriers were installed on the Memorial Bridge in Augusta, Maine (United States), Muenster Terrace in Bern (Switzerland) and the Bloor Street Viaduct in Toronto (Canada), respectively. Isaac and Bennett [22] and Skegg and Herbison [31] reported the same ‘reduction to zero’ finding when access was blocked to Beachy Head in Sussex (United Kingdom) and Lawyers Head Cliff in Dunedin (New Zealand). Bennewith and colleagues [17,18] also reported substantial decreases in the number of suicides (though not a complete elimination of them) following the erection of fencing on the Clifton Suspension Bridge in Bristol (United Kingdom). The fencing was accompanied by an expansion of the role of bridge staff to include monitoring of incidents, and the installation of CCTV cameras (see below). Lester [24] and O’Carroll and Silverman [27] reported similar findings to those of Bennewith and colleagues [17,18] when they independently examined data on suicides before and after modifications to the Ellington Bridge in Washington, DC (United States). Law et al. also noted a significant decrease in suicides following the introduction of platform screen doors on the Hong Kong underground railway system (Hong Kong) [23]. Conversely, when barriers on the Grafton Bridge in Auckland (New Zealand) were removed for aesthetic reasons, Beautrais and colleagues observed an increase in suicides [15,16]. Replacement of the original barriers by new ones with an improved design was followed by a decrease in suicides [15,16].

Eight of the above studies made some attempt to examine whether the reductions in suicide at the sites in question were associated with any commensurate increases in suicide at alternative sites in the given city. Of these, seven demonstrated that the number of suicides from other sites remained the same or decreased for the total population [23,24,27-29], or for males (who account for the majority of suicides by highly lethal methods, such as jumping from a height) [17,18]. However, Sinyor and Levitt [30] found no reduction in the overall numbers of suicides by jumping in Toronto during their respective study periods, suggesting that some substitution may have occurred.

Pelletier [28] and O’Carroll and Silverman [27] explored the substitution phenomenon further, and considered whether the observed reductions in suicides at the means-restricted sites equated to decreases in rates of suicide by any method (that is, not just jumping) in Augusta and Washington DC, respectively. Both of these studies identified small decreases in overall suicide rates following the means-restriction interventions.

### Encouraging help-seeking

Three studies have examined the effectiveness of installing signs and telephones at specific hotspots as a way of encouraging suicidal individuals to seek help [21,25,33]. King and Frost [21] evaluated an intervention that involved the placement of signs providing contact details for the Samaritans placed in car parks in the New Forest in Hampshire (United Kingdom). Lester [25] evaluated crisis emergency telephones on the Sunshine Skyway Bridge in St Petersburg, Florida (United States) which were installed at the same time as the introduction of a police presence on the bridge (see below). Wong et al. [33] evaluated an integrated community-based program of initiatives designed to reduce suicides by visitors to Cheung Chau (Hong Kong), an island where a number of people had taken their own lives by charcoal burning in rented holiday flats. This included, among other things, the introduction of a 24-hour telephone hotline service to support people in emotional distress, and the provision of hotline numbers in all holiday flats.

All three studies showed reductions in suicides at the specific sites following the introduction of the intervention [21,25,33]. King and Frost [21] and Wong et al. [33] examined patterns of suicide at comparison sites (that is, at car parks with no signs in the New Forest, and at other Hong Kong islands, respectively) and found no change over the relevant observation period. King and Frost [21] also explored the impact of the reduction at the intervention sites on the overall number of suicides in the district and found that this decreased, suggesting that substitution had not occurred.

### Increasing the likelihood of intervention by a third party

Three studies have explored the extent to which increasing the likelihood of intervention by a third party at hotspots can reduce suicide at these sites [17,18,25,33]. Two of these studies – by Lester [25] and Wong et al. [33] – have already been mentioned. They considered the impact of a range of activities on suicides from the Skyway Bridge and on Cheung Chau Island, respectively. This included a police presence on the former, and gatekeeper training and suicide patrols on the latter. Both studies reported positive findings in terms of reductions in numbers of suicides [25,33].

The third study, by Bennewith and colleagues [17,18], was also mentioned earlier. These authors examined an intervention that primarily involved the installation of barriers on the Clifton Suspension Bridge, but also included other components, namely an expansion of the role of bridge staff to include monitoring of incidents, and the installation of CCTV cameras. As noted, the intervention was associated with a reduction in suicides. The number of incidents remained stable, but bridge staff were more likely to be involved in incidents.

### Providing guidance on responsible media reporting of suicide

A single study has considered whether providing guidance on responsible reporting of suicide at a hotspot can lead to reductions in suicide at that site, although the authors have strengthened the evidence by adding further data and conducting a follow-up analysis some years after they did the original work [19,20,26,32]. Etzersdorfer and Sonneck [19,20], Sonneck et al. [32] and Niederkrotenthaler and Sonneck [26] found that completed and attempted suicide on the Vienna underground railway system (Austria) rose significantly in the latter part of the pre-intervention period (when sensationalist reporting of suicide was common). They then observed that, following the introduction of guidelines on responsible reporting, suicidal acts dropped dramatically to a level that has been sustained since [19,20,26,32]. There was some evidence that this contributed to an overall reduction in the national suicide rate over time [19,20,26,32].

## Discussion

### Interpreting the findings

Our review represents a consolidation of current knowledge about preventing suicides at hotspots, and an identification of gaps in that knowledge. Our starting point was the research question, ‘*What interventions are available to reduce suicides at hotspots, and are they effective?’* The answer to the first part of this question is relatively straightforward. Four main approaches have been used at suicide hotspots: (a) restricting access to means (through installation of physical barriers); (b) encouraging help-seeking (by placement of signs and telephones); (c) increasing the likelihood of intervention by a third party (through surveillance and staff training); and (d) encouraging responsible media reporting of suicide (through guidelines for media professionals).

The answer to the second part of the question is more complex. The strongest evidence for effectiveness comes from studies that have looked at restricting access to means through the installation of barriers at jumping sites and on railway networks. This body of evidence consistently suggests that these measures are associated with a reduction in suicides at these sites because they limit access or make it difficult to perform suicidal acts. In the main, the evidence also suggests that restricting access to means at one site does not drive suicidal individuals to seek alternative locations, thereby shifting the problem elsewhere. There are also indications that reducing suicides by a particular method does not lead to substitution of different methods; instead it may have a positive impact on the overall suicide rate. The apparent effectiveness of installing barriers at suicide hotspots is consistent with the broader literature on restricting access to means as a population-level suicide prevention strategy. Reviews by Mann et al. [34] and Beautrais et al. [35] suggest that this is one of very few approaches for which there is strong evidence of effectiveness. The theory behind restricting access to means is that it may ‘buy time’ for the individual to reconsider his or her actions, particularly in situations where these actions are associated with impulsivity or ambivalence [36-38].

Beyond this, the evidence is weaker. The evidence for the effectiveness of interventions designed to encourage suicidal people to seek help (for example, crisis telephone lines) and interventions designed to increase the likelihood of intervention by a third party (for example, suicide patrols and CCTV) is limited. Relatively few studies have looked at their impact. Those which have done have tended to examine these strategies in the context of broader suicide prevention programs at given hotspots, and this has made their independent influence difficult to evaluate. It is fair to say, however, that they show sufficient promise regarding reducing suicides at hotspots to be worthy of further testing. Different models (for example, crisis telephone lines which link directly to mental health services [39]) may be worth exploring.

The provision of guidance on how to report suicides at hotspots has also been subject to limited testing. Only one study has evaluated this strategy specifically. Although this study was well-designed and covered a lengthy observation period, it was restricted to a single setting in a single country. The study suggested that supporting journalists and editors in responsible reporting led to a decrease in suicidal acts on the rail network in question, and that this translated into a genuine overall reduction in suicides nationally. This is consistent with the literature on responsible reporting of suicide more generally (not just in the context of suicide hotspots), which suggests that guidelines can be effective in modifying the behaviour of media professionals and that this, in turn, can minimise imitation suicides [40-42].

### Future directions

Further research in this area is clearly warranted, and good quality evaluations of the latter three types of interventions are particularly necessary. These evaluations should adopt the strongest designs possible in the context of implementing interventions at hotspots. Recognising that these are not likely to be randomised controlled trials, suicide prevention researchers should draw on other areas of public health which have grappled with the issue of what constitutes sufficiently good evidence of effectiveness [43,44], and on the broader evaluation literature which stresses the importance of underpinning evaluations with sound program theory [45]. Triangulation of data from multiple sources is desirable, and might include both quantitative and qualitative data collection approaches. For example, ecological data (on pre- and post-intervention suicides from the site) could be combined with individual-level data (on the decision-making processes of those who sought help as a result of the intervention) where this is practically and ethically possible. Selecting comparison sites is also advantageous if this is feasible, particularly if the intervention can be rolled out across sites in a staggered fashion [46]. Likewise, examining the dose–response effect of gradually introducing the different components of a multi-faceted intervention may help to tease out the extent to which each individual component is effective. A consideration of cost-effectiveness is also important.

The above approaches to the reduction of suicides at known hotspots have been deemed to constitute current best practice, and have been advocated as part of a suite of potential measures in guidelines developed in England in 2006 (and translated into Japanese in 2007) [13], Scotland in 2012 [47] and Australia in 2012 [48]. This is appropriate under the circumstances; the need to strengthen the evidence base regarding interventions at hotspots should not prevent us from taking action in the immediate term, and the interventions considered in this review certainly show some promise. However, there is an onus on those who are responsible for funding and implementing these interventions to monitor them carefully to ensure that they are achieving their potential as effective suicide prevention strategies and are not having unintended consequences. Partnering with suicide prevention researchers with expertise in evaluation may be one way to do this.

### Limitations of the review

The current review has several limitations which must be acknowledged. We adopted as comprehensive a search strategy as we could, but resource constraints meant that we only had the capacity to search one database, we did not look for grey literature or conference abstracts, and we did not contact any authors for additional information. We did search two other databases (PsycINFO and Scopus) for a subsequent meta-analysis of studies on means restriction at hotspots and we found no additional papers [49]. Nonetheless, it is possible that some papers may have been missed. Publication biases may have operated, such that positive findings about particular interventions were more likely to have appeared in print than negative ones. It is also worth noting, however, that there would be instances where interventions have been implemented, with an effect, and not reported. This is particularly likely to be the case with barriers on bridges, which are not uncommon and may lead to reductions in suicides, but may not be reported in the scientific literature.

It was not always possible to determine the exact nature of the intervention. This was not so much a limitation of the review per se, but rather a limitation of the original studies. This limitation may have been a particular problem for the studies of restricting access to means, which tended to be reported as evaluations of a stand-alone intervention (the installation of barriers). Of these nine studies, only the one by Bennewith and colleagues [17,18] provided detail of additional activities that were put in place alongside the barriers on the Clifton Suspension Bridge. A commentary on the study by Sinyor and Levitt [30] by Sakinofsky [50] indicated that the barriers at the Bloor Street Viaduct were complemented by a telephone crisis service, but this was not evident from the original paper. This may not have been an isolated case. The upshot of this is that impacts may have been attributed to a single intervention that in reality were at least in part due to other activities that ran alongside it.

These limitations were compounded by more basic difficulties of definition. Although we have provided a definition of the term ‘hotspot’, in practice this may have been applied fairly loosely across studies, with the result that different authors would use different thresholds to deem a site a hotspot. Similarly, the term ‘intervention’ is not universally understood. Some of the activities classified as interventions were in fact opportunistic activities (e.g., road closures), other interventions within the same group undoubtedly varied in scope and scale, and still others, as noted above, were delivered as part of a suite. Finally, the term ‘effectiveness’ must be interpreted in the light of the evidence available, with due acknowledgement being given to the study design issues mentioned above.

## Conclusions

Notwithstanding the above limitations, we believe that our review demonstrates that there is consistent and relatively strong evidence that reducing access to means through the installation of barriers can be effective in averting suicides at hotspots and does not lead to substitution effects. The evidence is weaker for the other approaches that have been used, namely encouraging help-seeking, increasing the likelihood of intervention by a third party, and providing guidance on responsible media reporting of suicide, although they all show promise. This picture is consistent with the broader literature on suicide prevention, which suggests that there is good evidence that restricting access to means can work and that the majority of other interventions require further testing. Our review adds to this literature by focusing on the specific suite of interventions that have been used at suicide hotspots.

There is often community resistance to restricting access to means, despite this being the approach for which there is the greatest evidence of impact. Policy-makers and planners should be encouraged to recognize the demonstrated benefits of this approach, because they will often be faced with arguments of aesthetics and up-front costs which mean that they are likely to preferentially choose the other approaches. The other approaches may be useful too, but they clearly require further testing. More well-designed intervention studies are needed to strengthen this evidence base.

## Competing interests

The authors have no competing interests to declare.

## Authors’ contributions

JP, GRC, CO and JR were responsible for the original conceptualisation and design of the review, and AL, MW and YTDC helped to refine its parameters. GRC conducted the literature search and retrieved all potential articles. GRC, AL and JR screened abstracts and full text articles to arrive at the final pool. GRC and AN prepared early drafts of the paper, and all authors commented on and refined these drafts. All authors read and approved the final manuscript.

## Pre-publication history

The pre-publication history for this paper can be accessed here:

http://www.biomedcentral.com/1471-2458/13/214/prepub
